# Variations of cannabis-related adverse mental health and addiction outcomes across adolescence and adulthood: A scoping review

**DOI:** 10.3389/fpsyt.2022.973988

**Published:** 2022-10-10

**Authors:** Navdeep Kaur, Gabriel Bastien, Lea Gagnon, Johann Graham, Violaine Mongeau-Pérusse, Hamzah Bakouni, Florence Morissette, Camille Theriault, Benedikt Fischer, Didier Jutras-Aswad

**Affiliations:** ^1^Centre de Recherche du Centre Hospitalier de l’Université de Montréal (CRCHUM), Montréal, QC, Canada; ^2^Department of Psychiatry and Addiction, Faculty of Medicine, Université de Montréal, Montréal, QC, Canada; ^3^Faculty of Medicine, Université Laval, Quebec City, QC, Canada; ^4^School of Population Health, Faculty of Medical and Health Sciences, University of Auckland, Auckland, New Zealand; ^5^Department of Psychiatry, University of Toronto, Toronto, ON, Canada; ^6^Centre for Applied Research in Mental Health and Addiction, Faculty of Health Sciences, Simon Fraser University, Vancouver, BC, Canada; ^7^Department of Psychiatry, Universidade Federal de São Paulo (UNIFESP), São Paulo, Brazil

**Keywords:** addictive behavior, adolescent, adult, cannabis, mental health

## Abstract

**Introduction:**

Evidence supporting associations between cannabis use and many health outcomes is growing, however it remains unclear how such associations vary across the lifespan. We therefore aim to answer the following questions: (1) Are the risks of cannabis’s adverse effects on mental health and addiction-related outcomes different in adolescents than in adults? (2) What are the relationships between these cannabis’s adverse effects and (a) an individual’s age at first cannabis use, (b) age at assessment, and (c) duration of cannabis use?

**Methods:**

We searched Medline, Embase, CINAHL, and PsychINFO from inception to 18 October 2021. Two reviewers independently screened studies and descriptively synthesized results.

**Results:**

We included 140 studies. Cannabis effects on mental health and addiction-related outcomes were worse in adolescents, early cannabis initiators and cannabis users who consumed for longest periods. Evidence of worse long-term adverse effects in adolescents was substantial for psychosis, cannabis, and nicotine use disorders; mixed for depression, suicidality, other substance use and disorders; and limited for anxiety. Additionally, acute cannabis exposure had the opposite trend with adults more often reporting adverse effects than adolescents.

**Conclusion:**

The available evidence suggests that cannabis use should be delayed as late as possible in adulthood and shortened in duration across the lifespan to decrease the risk of negative outcomes, while emphasizing the need for adapted harm reduction approaches. This scoping review provides evidence on the role of age and duration of exposure as determinants of cannabis-related adverse effects, which may inform prevention and harm reduction strategies.

**Systematic review registration:**

https://doi.org/10.17605/OSF.IO/BYG72

## Introduction

Around the world, almost 200 million people consumed cannabis in the past year, making it the most used psychoactive substance after nicotine and alcohol (1–3). Cannabis use prevalence is particularly high among adolescents and young adults (3). Consumed for recreational purposes and its therapeutic properties, cannabis can also adversely impact users’ health (4, 5). Cannabis exposure has been associated with a myriad of physical, mental, and psychosocial adverse health outcomes affecting all age groups (5). Notably, early cannabis initiation while the brain is still developing has been hypothesized to distinctly predispose adolescents to detrimental effects and increase risks specifically for mental health, cognitive and addiction problems (6). For example, adolescent cannabis use has been associated with psychiatric disorders such as schizophrenia (7–12), depression, and suicidal behavior (13). The probability for cannabis users to transition to cannabis use disorder (CUD) range between 9 and 27%, depending on the sample population, diagnosis definition and age of exposure onset (14, 15). Some evidence also suggests that early cannabis consumption may lead to the use of other substances (16, 17).

With the prevalence of cannabis use on the rise in many contexts and some jurisdictions liberalizing controls (including legalization) for recreational use (18), the establishment of a strong evidence base is needed to guide best public health strategies, harm reduction interventions, and policies. Existing initiatives have traditionally employed a precautionary approach assuming higher risks of harms in youth than in adults, thus suggesting broadly to avoid early and generally delay exposure to cannabis. While the body of evidence on associations between cannabis use and health outcomes is progressing, however, most of the existing literature reviews on cannabis harms on mental health and addiction in humans focused on a narrow set of mental health outcomes (7, 19) or on specific age groups (20). Consequently, it remains unclear how such associations may vary across the lifespan and whether they do similarly for all outcomes. To map the existing evidence as well as to identify any knowledge gap, we conducted a scoping review to answer these research questions (RQs): (1) Are the risks of cannabis’s adverse effects on mental health and addiction-related outcomes different between adolescents and adults? (2) What are the relationships between these cannabis’s adverse effects and (a) the user’s age at first cannabis use (b) participant’s age at assessment, and (c) duration of cannabis use?

## Methods

We followed the Joanna Briggs Institute (21) scoping review methodology and the Preferred Reporting Items for Systematic Reviews and Meta-Analyzes extension for Scoping Reviews (PRISMA-ScR) guidelines (22) to prepare our prospectively published protocol (23).

### Eligibility criteria

Human studies were included if they: (i) reported adverse mental health or addiction outcome(s) related to cannabis use, (ii) reported relationship(s) between cannabis’s adverse mental health or addiction outcome(s) and cannabis use onset or duration, (iii) included adolescents (<18 years old) and adults (≥18 years old), (iv) were published in peer-reviewed journals, and (v) were available in English, French, or Spanish. Editorials, letters, research protocols, and commentaries were excluded. To retrieve a full text that could not be obtained through institutional holdings, a librarian, the author, or the journal editor was contacted.

### Data sources and search strategy

We searched for publications in three main electronic databases (MEDLINE, Embase, and PsychINFO) from inception to 27 October 2020. However, due to unforeseen circumstances caused by the COVID-19 pandemic, our scoping review got bit delayed therefore, to ensure inclusion of latest publications, we updated our search strategy on 18 October 2021. We consulted a specialized librarian to develop and execute a specific search strategy for each database. The search concepts were: (i) cannabis, (ii) adolescents and adults OR age of onset or initiation, and (iii) adverse or negative effects OR mental health OR addiction. Supplementary Appendix 1 presents our first and updated search strategies for Medline that were adapted for other databases. Furthermore, we manually searched through the reference lists of all identified records for retrieving additional relevant studies.

### Study selection process

All citations were imported into the EndNote X9 software. A screening form was developed *a priori*. Distiller SR^®^ was used for data extraction and study selection. We followed a three-step study selection process. First, all duplicate publications were removed. Second, two reviewers (GB and VM, JG and CT/NK, FM and LG, NK, and HB) screened titles and abstracts with the screening form. Third, full texts meeting the inclusion criteria were reviewed and relevant studies were selected. Two independent reviewers (GB and VM, FM/JG and LG, NK, and HB) screened and extracted data from the full texts and a third reviewer (DJ-A/NK) resolved discrepancies between reviewers.

### Data charting and synthesis

From each study, the following information was extracted: first author’s name, publication year, country of study, study design, sample size, and cannabis use definition and findings. The main outcomes of interest were mental health, addiction and addictive behaviors related to cannabis use among adults and adolescents, relationships between cannabis’s adverse mental health and addiction effects AND (a) participant’s age at first cannabis use OR (b) participant’s age at assessment OR (c) duration of cannabis use. In this scoping review, the “participant’s age at assessment” is defined as the participant’s age at study participation. Data were synthesized descriptively, and study characteristics were presented in a tabular form including structured summaries of the study characteristics and findings.

## Results

### Search findings

**FIGURE 1:**
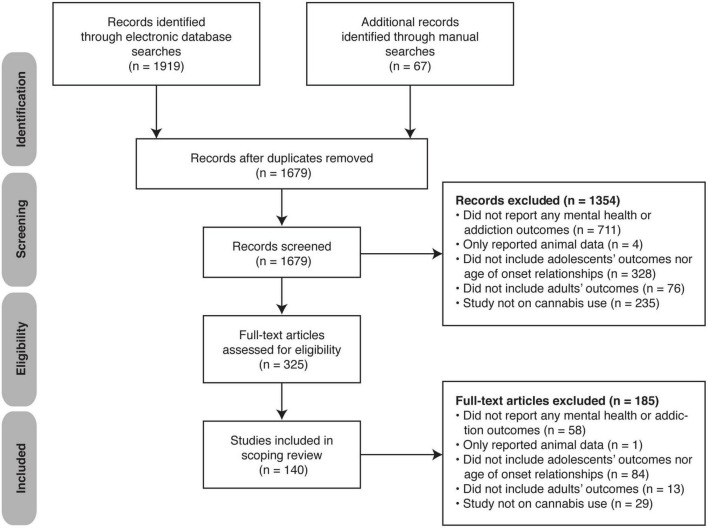
PRISMA flow diagram of the search and study selection process.

In total, 1986 studies (Medline *n* = 933; Embase *n* = 876; PsychINFO *n* = 110; and manual sources *n* = 67) were identified. After removing duplicates, 1,679 remained. Of these, 1,354 ineligible studies were excluded, and the remaining 325 full texts were reviewed. Finally, 185 studies were excluded leaving a total of 140 studies included in this scoping review (Figure 1).

### Characteristics of included studies

Among the 140 included studies, 135 were in English (9–13, 16, 20, 24–151) three in French (152–154), one in Spanish (155) and one was available both in French and English (7). There was one meta-analysis (88), one systematic review (7), two combined meta-analyses and systematic reviews (13, 20), 11 literature reviews (33, 61, 76, 86, 87, 90, 95, 110, 124, 136, 152), four randomized controlled trials (79, 100, 112, 134), 61 cohort studies (10, 16, 26, 28, 29, 32, 35, 36, 40–45, 47–50, 55, 60, 62, 64, 68, 69, 71, 74, 78, 80, 83, 92, 96, 98, 104, 106–109, 111, 116, 118, 119, 123, 125, 128, 130, 132, 133, 135, 138–147, 149–151), 52 cross-sectional studies (11, 12, 24, 25, 27, 30, 31, 34, 37–39, 46, 51–54, 57–59, 66, 67, 70, 72, 73, 75, 77, 81, 82, 84, 85, 89, 91, 94, 97, 99, 101–103, 105, 113, 115, 117, 120, 121, 126, 127, 131, 137, 148, 153–155), three repeated cross-sectional studies (93, 122, 129), one naturalistic study (56), two retrospective cohort studies (65, 114), and two case-control studies (9, 63). Characteristics of the included studies are presented in Supplementary Tables 1–4 and the main findings are described below.

### Main findings

Research question 1: Are the risks of cannabis’s adverse effects on mental health and addiction higher in adolescents compared with adults?

Supplementary Table 1 summarizes the 12 studies comparing cannabis’s adverse mental health and addiction effects between adolescents and adults.

(i) Psychotic symptoms

Two studies by Mokrysz et al. (100, 134) reported that adults acutely exposed to cannabis experienced more psychotic-like effects than adolescents. Kelley et al. (99) found that daily cannabis use in adolescents tripled and in adults doubled the rate of onset of psychosis. Albertella et al. (10) reported that younger frequent cannabis users showed increased negative schizotypy while older frequent users showed reduced negative schizotypy.

(ii) Anxiety

Mokrysz et al. (100) observed that adults acutely exposed to cannabis rated their anxiety higher than when exposed to placebo while adolescents reported no such difference.

(iii) Suicidality

Levine et al. (110) concluded adolescent cannabis users are at higher risk of later suicidality.

(iv) Cannabis use and cannabis use disorder

Adolescents reported using cannabis more often than adults (115). Four studies (24, 31, 91, 105) reported 1.3–2.5 increased odds of developing a CUD in adolescent compared with adult cannabis users. Mokrysz et al. found that adolescents felt less stoned, felt the drug less, wanted more cannabis following exposure (100) and scored lower on negative experience (77) compared with adults.

(v) Other substance use disorders

Wang et al. (102) concluded that the odds of co-occurring nicotine dependence greatly varied with age of cannabis use, reaching peak values during adolescence and late adulthood. Levine et al. (110) reported that adolescent cannabis users were at increased risk of addiction to several substances.

(vi) Other adverse effects on mental health

Hawke et al. (115) found that adolescent cannabis users were more likely to have an externalizing disorder such as attention deficit hyperactivity disorder than adult users. Levine et al. (110) indicated that adolescent cannabis users are at increased risk of psychiatric morbidity.

Research question 2: What are the relationships between cannabis’s adverse effects and (a) participant’s age at first cannabis use, (b) participant’s age at assessment, and (c) duration of cannabis use?

(a) Supplementary Table 2 summarizes the 115 studies on relationships between cannabis’s adverse mental health and addiction effects and participant’s age at first cannabis use.

(i) Psychosis and related disorders

Forty-four studies reported that early cannabis initiation was associated with psychotic outcomes, including earlier age of onset of psychosis (7, 9, 53, 61, 64, 65, 73, 75, 82, 97, 99, 101, 138), higher risks of psychotic symptoms (12, 38, 56, 66–68, 70, 71, 75, 94, 95, 103, 111, 116, 127, 131, 150) and greater severity of these symptoms (20, 61, 89), higher risks of psychosis (61, 86, 90, 131, 147), and higher risk of psychotic disorder (7, 59, 87, 95, 136, 152) compared with later initiation or non-use of cannabis. Two studies (73, 82) revealed that the onset of psychosis followed cannabis initiation on average 7–8 years after. Two studies (59, 89) associated the risk of cocaine-induced psychosis to an earlier age of cannabis use. A review (86) suggested that the higher risk of psychosis in early cannabis users was dose dependent. Eight studies (63, 80, 92, 97, 112, 116, 125, 128) found no association between psychosis outcomes and the age of cannabis initiation. Curran et al. (121) reported opposite results with older age of cannabis initiation associated with more psychotic symptoms.

(ii) Anxiety

Five studies (87, 106, 128, 142, 143) reported that early-age cannabis users had between two and three times the odds of anxiety disorders compared with non-users, while five studies (13, 68, 92, 132, 149) including a meta-analysis (13) and a cohort study (149) found no such association after adjusting for demographics and childhood adversities. Another study indicated that such increased risk was limited to early and frequent cannabis users (78). Late cannabis initiation was associated with a fourfold increase in odds of developing an anxiety disorder as compared with non-users (128) a finding supported by another team reporting similar probability (92). Of the four studies comparing the risk or incidence of anxiety disorders between early and late cannabis users, only one (96) found a fourfold increased odds while the other three (92, 107, 132) found no difference. Dragt et al. (71) reported that age at first cannabis use positively correlated with age of onset of anxiety symptoms while two other studies (67, 112) found no such correlation. Two studies (61, 87) concluded that early cannabis initiation is a risk factor for anxiety disorders for frequent cannabis users. Hosseini et al. (7) indicated that too low quality of evidence exists on anxiety for recommending a minimum age for cannabis use for preventing this outcome.

(iii) Depression

Fifteen studies (13, 29, 33, 37, 61, 68, 72, 87, 88, 98, 119, 139, 142, 143, 152) reported that early cannabis initiators had between 1.1 and 8.8 times the odds of depression compared with non-users, while eight studies (37, 48, 50, 67, 78, 98, 128, 149) reported similar odds, incidence or no association. Four studies (72, 128, 139, 150) found that late cannabis initiators had between 1.6 and 3.3 times the odds of depression compared with non-users while two studies (29, 119) reported similar odds. These increased odds of depression disappeared after covariates adjustment in three studies (37, 142, 143). Lynskey et al. (37) revealed that depression risk was increased only in dizygotic twins discordant for early cannabis use, but not in monozygotic twins, before confounders adjustment, indicating a genetic modulatory effect. Harder et al. (48) found 2.6 times increased odds of depression only in males with problematic cannabis use in adolescence compared with those without problematic cannabis use. Three studies (54, 96, 132) showed that early cannabis initiators were between 1.2 and 1.9 times more likely to develop depression compared with late initiators, while another study (26) found similar likelihood. Out of three studies (37, 67, 71, 112) assessing correlations, one (71) found a positive correlation between age of cannabis initiation and age of onset of depressed mood.

(iv) Suicidality

Five studies (13, 37, 104, 128, 142) including a meta-analysis (13) reported that early cannabis initiators had 1.5–4.2 times the odds of considering suicide and 1.7–8.7 times the odds of attempting suicide compared with non-users (13, 37, 88, 104). However, these relationships in some instances became non-significant after covariates adjustment (37, 128, 142) and two studies (50, 106) reported no association. Silins et al. (88) found that the higher risk of suicide attempts in early cannabis users depended on cannabis use frequency, with daily cannabis use having the highest odds. Late cannabis users had a similar risk of suicide ideation (104, 128) and suicide attempts (104) compared with never users. When comparing early with late cannabis users, the suicidality risk was increased twofold in the early users (132). Savage et al. (112) reported that age of cannabis initiation negatively correlated with suicide risk rating.

(v) Other cannabis use outcomes

Baggio et al. (83) reported higher proportions of early compared with late cannabis users who felt high, relaxed, laughed a lot, and did crazy things the first time they tried cannabis. Ellickson et al. (40) indicated that a younger age at cannabis initiation was associated with negative consequences such as concentration problems. A cross-sectional study (39) found that early-age cannabis users (<16 years old) had increased odds of problematic cannabis use than later-age users. Bravo et al. (120) observed that younger age at first cannabis use was associated with less reliance on cannabis protective behavioral strategies. A cohort study (144) reported that early cannabis use increased the likelihood of continued cannabis use in adulthood.

(vi) Cannabis use disorder

Five studies (34, 88, 108, 123, 128) found that, depending on use frequency, cannabis users starting in adolescence had between two and 300 times the odds of subsequent cannabis dependence compared with non-users. When controlling for covariates, these odds ratios remained significant and varied between two and 253 (34, 55, 88, 108, 128). Furthermore, rates of dependence were between four and 14 times higher in early-age cannabis users compared with never users (143). Two cohort studies (36, 107) and two cross-sectional studies (25, 85) reported similar risk of dependence in early-age compared with later-age cannabis initiators. Three cross-sectional studies (34, 85, 131) and three cohort studies (32, 69, 151) found 2.0–2.7 times increased risk of developing cannabis abuse or dependence in early compared with later-age cannabis initiators. Interestingly, each year older at first cannabis use reduced the odds of developing dependence by 11% (84). People who develop cannabis dependence are more likely to have a younger age of initiation than non-problematic cannabis users (46). The time from first cannabis use to cannabis dependence diagnosis increased from 28 years in cannabis initiators starting before age 13, to 47 years in initiators starting after age 19 (57). The increased risk of dependence among young cannabis initiators was further supported by three narrative reviews (95, 124, 152) and a cohort study (145). Four studies further associated early initiation with risky cannabis use (81), severe cannabis dependence (126), and CUD (148).

(vii) Other substances use

Four studies found that early cannabis users were more likely to consume tobacco than non-users (68) or late cannabis users (49, 103, 130). Three studies (44, 85, 96) supported this finding but only for daily tobacco use, and this association was non-significant after covariates adjustment in another study (149). Moore and Budney (27) reported a younger age at first cannabis use among tobacco smokers compared with non-smokers. Mixed evidence was found among the three studies (85, 93, 96) measuring alcohol use. Moss et al. (85) found no difference between early cannabis users and non-users for monthly and yearly alcohol use, while Buu et al. (93) noted an increased risk of heavy alcohol use for both early and late cannabis users compared with non-users. Few et al. (96) revealed that early cannabis users had twice the odds of regularly using alcohol compared with their late using co-twin. Stanley et al. (137) reported that while late cannabis users had 16 times the odds of misusing prescription drugs compared with non-users, early cannabis users had 47 times these odds. Early cannabis initiators had twice the odds of misusing prescription opioids compared with non-users (135), and nearly twice the risk of prescription opioid misuse compared with late users (129). This contrasts with Moss et al. (85) findings of similar prevalence of pain reliever misuse between early cannabis users and non-users. Hall et al. (95) reported that majority of the 17 studies reviewed associated early cannabis use with other illicit substance use. Early cannabis users had between two and 14 times the odds of using other drugs compared with non-users (34, 41, 42, 68, 88, 135). After covariates adjustment, these odds were increased to between two and 17 times (34, 62, 88, 135), or became non-significant (149). These results are in line with increased prevalence of a range of illicit drug uses among early cannabis users compared with non-users (47, 85), especially in frequent cannabis users (143). Early cannabis users were sometimes as likely (39, 79) and sometimes more likely (96, 103) to use illicit substances than late users. Finally, the age of cannabis initiation negatively correlates (medium effect size) with illicit drug use frequency (40).

(viii) Other substance use disorders

Early cannabis users were more likely to develop nicotine dependence than non-users (45, 55, 128) or late users (44, 45, 131). However, two studies reported no difference either in this risk between early cannabis users and non-users (85) or in the incidence of nicotine use disorder in early versus late cannabis initiators (107). The risk of developing an alcohol dependence was also higher for early cannabis users compared with non-users (55, 128, 143) or late users (36). However, this relationship sometimes became non-significant after covariates adjustment (55, 143). Another study (107) found similar incidence of alcohol use disorder in early- and later-age cannabis users. When controlling for confounders, early cannabis initiators had between 2 and 66 times the odds of illicit substance use disorder (SUD) (29, 128) or drug abuse (62) and twice the prevalence of illegal drug dependence (85) compared with non-users. The review by Dervaux et al. (152) further supported these results. Four studies (34, 118, 131, 145) found that the risk of illicit drug use or dependence depended on the age at first cannabis use and the type of other drug involved. For example, it was the highest in cannabis initiators starting before age 13 and became non-significant after age 15 (118). Moreover, it was higher for cocaine/stimulants, and opioids (34) but similar for methamphetamines (131) and sedatives (34).

(ix) Other mental health outcomes

As reviewed by Rubino et al. (76) three studies reported that early cannabis initiation increased the odds of psychological distress (103), subclinical psychotic experience (66), or non-suicidal injury (96) compared with later cannabis initiation. When compared with non-users, early and frequent cannabis use increased the odds of anxiety and depression two to threefold while late and frequent cannabis use increased it twofold (43). Estrada et al. (11) reported a positive correlation between age at first use and age of onset for psychiatric illness. Shah et al. (114) found that early cannabis initiation predicted progression to a cannabis-induced psychotic or mood disorder. Eight studies (35, 36, 40, 67, 92, 113, 149, 155) found no relationship between age of cannabis initiation and psychiatric disorders.

(b) Supplementary Table 3 summarizes the 12 studies on relationships between cannabis’s adverse mental health and addiction effects and participant’s age at assessment.

(i) Depression and anxiety

The associations between cannabis use and depression symptoms differed with age (133). When depression and anxiety were measured together, however, Meier et al. (133) found no evidence of an association with age. When assessed separately, one cohort study (74) confirmed that cannabis use at younger age was associated with increased depressive symptoms compared with older age. Conversely, although Patton et al. (146) did not directly compare age groups in their cohort, the association between daily cannabis use and depression and anxiety during adulthood was stronger for past-year adult use than for adolescent use in women only (similar in males).

(ii) Suicidality

Fergusson et al. (141) showed that the strength of association between cannabis use frequency and suicidal ideations and attempts decreased with increasing age (14–21 years old).

(iii) Cannabis use and cannabis use disorder

Fergusson et al. (140) indicated that the cumulative risk of cannabis dependence increased gradually from 0% at age 16 to 9% at age 21. Wagner et al. (30) showed a moderate risk for developing CUD following early cannabis use. Madruga et al. (148) indicated that odds of current or past-year cannabis use decreased with progressing age. Chen et al. (144) reported that early age is a predictor of ongoing cannabis use. Padovano et al. (117) reported that adolescents experienced a greater subjective high experience than young adults.

(iv) Other substances use and substance use disorders

Two studies (16, 62) indicated that the odds of other illicit substance use following cannabis use declined with increasing age. One study (16) confirmed similar significant associations for other substance use. Another study (155) reported that younger age is associated with SUD among cannabis users. Finally, Fergusson et al. (141) showed that the strength of association between cannabis use frequency and illicit drug use decreased gradually with increasing age (between ages 14 and 21).

(v) Other mental health outcomes

A cross-sectional study (155) showed that age was associated with the presence of Axis I psychiatric disorders other than SUD but not with Axis II disorders among cannabis users.

(c) Supplementary Table 4 summarizes the 18 studies on relationships between cannabis’s mental health and addiction adverse effects and duration of cannabis use.

(i) Psychosis and related disorders

Two studies (52, 60) found that a cannabis use duration of more than 5 or 6 years increased the odds of experiencing psychosis twofold compared with a shorter duration or no cannabis use. Two studies (97, 147) found no correlation between cannabis use duration and age of onset of psychotic disorder (97) nor transition to psychosis (147).

(ii) Anxiety

Four (58, 94, 128, 133) out of the five studies (58, 94, 128, 133, 153) focusing on anxiety found a positive relationship with cannabis use duration. Cannabis users consuming for at least 11 years, between 2 and 10 years, and for 1 year or less had respectively 2.8, 2.3, and 1.6 times the odds of anxiety compared with non-users (58). Similarly, weekly cannabis users consuming for 16 years had 2.1 (2.5) times the (adjusted) odds of anxiety compared with non-users whereas those who weekly used for 8 years had 2.3 (2.8) times these odds (128). Although Cloak et al. (94) did not measure cannabis use duration *per se*, they found positive small-effect size correlations between cumulative lifetime quantity of cannabis and anxiety symptoms or phobic anxiety. Furthermore, Meier et al. (133) indicated that each additional year of weekly cannabis use increased the risk of anxiety and depression problems, when measured together. Another cross-sectional study (153) found no correlation between cannabis use duration and anxiety.

(iii) Depression and suicidality

Four studies associated a higher risk of depression (58, 72, 122, 133), one study associated depressive symptoms (153) and one study associated suicidal ideation (128) with long cannabis use duration. Two studies (58, 72) indicated that cannabis users who consumed cannabis for more than 11 years had nearly three times the odds of depression compared with non-users, whereas those who used between 2 and 10 years had twice these odds. Chabrol et al. (153) also reported a positive correlation between the depression score and cannabis use duration. Similarly, Meier et al. (133) indicated that each additional year of weekly cannabis use slightly increased the risk of depression. Conversely, Han et al. (122) reported a decreased depression prevalence for longer cannabis use duration (>3 years) than shorter duration (1–2 years) among adolescents.

(iv) Cannabis use disorder

Five (24, 84, 128, 154, 156) studies found an increased prevalence or risk with longer cannabis use duration. Von Sydow et al. (28) indicated that cannabis users develop cannabis abuse and dependence on average 2.0- and 2.4-years following initiation, respectively. Han et al. (122) reported an increased prevalence of CUD among adolescents and adults (adjusted prevalence of CUD in adolescents increased from 10.9 to 20.6% and in adults from 5.6 to 10.5% between the first and the fourth year of cannabis use).

(v) Other substance use disorders

Two cross-sectional studies (155, 156) and two cohort studies (128, 149) confirmed an increased prevalence or risk of SUD with longer cannabis use duration whereas Degenhardt et al. (51) found the opposite association. Han et al. (122) reported that after long cannabis use periods, both adults and adolescents developed other SUDs related to nicotine, alcohol, cocaine, hallucinogen, tranquilizers/sedatives, and opioids.

(vi) Other mental health outcomes

Three studies found positive small-effect size correlations between cannabis use duration and borderline personality disorder (153), and obsessive-compulsive scores (94). Cuenca-Royo et al. (155) found similar odds of psychiatric diagnosis in cannabis users consuming for 5–7 years compared with those consuming from 1 to 4 years.

## Discussion

Our scoping review’s results indicate that cannabis use is overall associated with higher likelihood of adverse mental health and substance use outcomes among adolescents, early cannabis initiators and cannabis users who consumed for longest periods. The strength of evidence varied based on the types of mental health and addiction outcomes. Substantial evidence was found for psychotic disorders, as well as cannabis and nicotine use disorders. Mixed evidence was obtained for depression and suicidality, other substance use, and other SUDs while it was limited for anxiety. Acute cannabis exposure led to the opposite trend with adults more often reporting adverse effects compared with adolescents. While our findings are overall consistent with three other recent reviews (7, 13, 95) on specific outcomes (i.e., psychosis, depression, other substance use, and suicidality) of cannabis exposure, we identified several knowledge gaps in the literature with some inherent limits and strengths in this scoping review.

Nearly half of the studies evaluating the effect of age of use initiation on cannabis-related harms compared early cannabis users with non-users instead of later-age users. Consequently, it was impossible to disentangle the effects of cannabis use from age at first use other than by comparing results with those obtained in similar studies conducted in older samples. Moreover, studies divided their age groups using different age categories, and most of the included studies measured cannabis consumption using self-report data. This type of measurement may be prone to recall and social desirability biases. More importantly, it prevents from accurately identifying exposure to specific cannabinoids (i.e., tetrahydrocannabinol and cannabidiol) and the level of such exposure. Future research should use complementary biological sampling to improve measurement of cannabis and cannabinoid exposure, like did few authors (11, 100, 113, 114, 131, 134). This is even more important with the continuously changing concentrations of tetrahydrocannabinol and cannabidiol especially in cannabis products obtained from the unregulated market (157–159). These ongoing changes in cannabis composition and potency also highlight the need for repeated assessments of the risks of cannabis use, which may fluctuate over time, as different products are made available to consumers across all age groups.

Age of cannabis use initiation and duration of cannabis use were main factors influencing the magnitude of cannabis-related harms. Other important contributors and potential effect moderators include cannabis potency (160), use frequency (161), familial medical history (162), and peer influence (163, 164). However, not all studies controlled for these potential confounders and among those who did, the associations were sometimes non-significant. This suggests that young age, early initiation, and longer duration of cannabis use represent only some of a complex array of risk factors that contribute to potential adverse outcomes of cannabis exposure. Overall, there was no clear evidence of a specific age of use at which cannabis-related harms could be avoided; such threshold would likely vary according to specific outcomes of interest. This prevents us from advising an age limit for “safe” cannabis consumption and highlights the challenging nature of such efforts. Notwithstanding the limitations of the available literature, it is reasonable to suggest that delaying cannabis consumption as late as possible and limiting the duration of use could decrease the risk of both short- and long-term adverse effects, aligned with the recommendations of the Lower Risk Cannabis Use Guidelines (165). Equally important, and as has been proposed by others, efforts are required to further standardize measurement of cannabis exposure, outcomes to prioritize, and potential confounders to facilitate knowledge synthesis.

Beyond the restrictions of the available literature as described above, this scoping review has its very own strengths and limitations. One of the key strengths of the present scoping review is that we used a broad search strategy and included highly heterogeneous study designs and measurement methods. This allowed us to obtain a wide overview of cannabis harms on mental health and addiction. Other outcomes related to mental health such as cognitive function, however, were outside the scope of this review and merit further attention. Also, we limited our selection to studies published in English, French, and Spanish. This could have introduced a small language bias that, however, seems to be unlikely to change our conclusions. Finally, in this review we broadly used the term “adulthood,” which, at least in theory, included “senior age.” Future research and knowledge synthesis efforts should pay specific attention to that age group to determine if and how some outcomes may specifically vary among older adults.

## Conclusion

In conclusion, age of exposure seems likely to modulate cannabis use-related mental health and addiction outcomes. Cannabis’ adverse effects on the long-term outcomes tended to be generally worse in adolescents, early cannabis use initiators and cannabis users who consumed for long periods. Thus, delaying cannabis use initiation to as late as possible in young adulthood and limiting cannabis use to short periods could decrease the risk of adverse cannabis use consequences. Using a harm reduction perspective, we advocate for providing youth with nuanced and accurate information on potential effects of cannabis use and develop interventions to promote safer cannabis consumption practices, taking into consideration specific risks associated with early-age cannabis use, which are not the same for all outcomes. Finally, we recommend that future research efforts on age-specific cannabis harms account for important confounding factors such as frequency and potency of cannabis consumed, and other key individual and environmental factors.

## Data availability statement

The original contributions presented in this study are included in the article/Supplementary material, further inquiries can be directed to the corresponding author.

## Author contributions

DJ-A and NK: study conceptualization and supervision. NK: methodology. GB, LG, JG, VM-P, NK, HB, FM, and CT: study selection. GB, LG, JG, NK, HB, and VM-P: data charting and synthesis. NK, JG, LG, and GB: original manuscript draft writing. LG, GB, HB, NK, DJ-A, and BF: data interpretation. NK, GB, LG, JG, VM-P, HB, FM, CT, BF, and DJ-A: reviewing and editing of the manuscript. DJ-A: funding acquisition. All authors contributed to the article and approved the submitted version.
