# Supplementary material for: Variations of cannabis-related adverse mental health and addiction outcomes across adolescence and adulthood: A scoping review
Source: Front Psychiatry. 2022 Oct 10;13:973988. doi: 10.3389/fpsyt.2022.973988 (PMC9590692; doi:10.3389/fpsyt.2022.973988)
Supplement: Supplementary file 2 [file Data_Sheet_1.docx]

# Supplementary Material

## Appendix 1: First Search Strategy for Medline

Ovid MEDLINE(R) and Epub Ahead of Print, In-Process & Other Non-Indexed Citations, Daily and Versions(R) <1946 to October 27, 2020>

| 1 | cannabis/ | 9303 |
| --- | --- | --- |
| 2 | exp “Marijuana Use”/ | 5823 |
| 3 | Hempa/ | 101 |
| 4 | (Cannabis or Marihuana* or Marijuana* or Hemp or Hemps or Ganja or Ganjas or Hashish or Hashishs or Bhang or Bhangs or Smoking Blunt*). ti. | 15726 |
| 5 | (Cannabis or Marihuana* or Marijuana* or Hemp or Hemps or Ganja or Ganjas or Hashish or Hashishs or Bhang or Bhangs or Smoking Blunt*). ab. /freq=3 | 11374 |
| 6 | (weed* adj3 (smok* or use* or using or abus* or dependen* or disorder*)). tw. | 551 |
| 7 | (hempa or hmpa or Hexametapol or Hexamethylphosphor*). tw. | 489 |
| 8 | 4 or 5 or 6 or 7 | 19271 |
| 9 | limit 8 to (“in data review” or in process or publisher or “pubmed not medline”) | 3528 |
| 10 | 1 or 2 or 3 or 9 | 17645 |
| 11 | adolescent/ | 2044410 |
| 12 | (adolesc* or juvenile* or teen* or youth* or pubescen* or preadolesc* or prepubesc* or preteen* or tween* or (young* adj3 (adult* or male? or female? or wom? n or lady or ladies or man or men or person? or people)) or ((emerging or early) adj1 (adult* or male? or female? or wom? n or lady or ladies or man or men or person? or people)) or (work* adj2 age?)). tw. | 664525 |
| 13 | 11 or 12 | 2385051 |
| 14 | adult/ or young adult/ | 5157182 |
| 15 | adult*. tw. | 1273508 |
| 16 | 14 or 15 | 5915629 |
| 17 | 13 and 16 | 1636109 |
| 18 | age of onset/ | 38071 |
| 19 | age factors/ | 454305 |
| 20 | (age adj3 (factor or initiation or onset)). tw. | 49904 |
| 21 | 17 or 18 or 19 or 20 | 2020092 |
| 22 | 10 and 21 | 3922 |
| 23 | ((adverse or deleter* or negative or harmful or acute) adj5 (effect* or outcome* or result* or impact* or symptom*)). tw. | 660451 |
| 24 | Depressive Disorder/ | 73221 |
| 25 | Depression/ | 121205 |
| 26 | exp Mental Disorders/ | 1252543 |
| 27 | Anxiety/ | 82536 |
| 28 | exp Psychotic Disorders/ | 52521 |
| 29 | substance-related disorders/ or psychoses, substance-induced/ | 100533 |
| 30 | schizophrenia/ or schizophrenia, paranoid/ | 104402 |
| 31 | Marijuana Abuse/ | 6393 |
| 32 | or/24–31 | 1363494 |
| 33 | (ae or de or pp). fs. | 5872096 |
| 34 | 32 and 33 | 340247 |
| 35 | 23 or 34 | 975808 |
| 36 | 22 and 35 | 992 |
| 37 | limit 36 to (english or french) | 940 |
| 38 | (Animals/ or Models, animal/ or Disease models, animal/) not Humans/ | 4716518 |
| 39 | ((animal or animals or canine* or cat or cats or dog or dogs or feline or hamster* or lamb or lambs or mice or monkey or monkeys or mouse or murine or pig or pigs or piglet* or porcine or primate* or rabbit* or rats or rat or rodent* or sheep* or veterinar*) not (human* or patient* or women or men)). ti, kf, jw. | 2395729 |
| 40 | 38 or 39 | 5135105 |
| 41 | 37 not 40 | 932 |
| 42 | limit 41 to (case reports or comment or congress or editorial or interview or introductory journal article or lecture or letter or news or newspaper article or overall or patient education handout) | 43 |
| 43 | 41 not 42 | 889 |
| 44 | limit 43 to abstracts | 799 |

**Note:** On January 21^st^, 2021, this search was repeated to include articles in Spanish as well. Search strategy retrieved only five Spanish articles in Medline making total number of articles retrieved on Medline n=814.

## Updated Search Strategy for Medline [OVID]

**Ovid MEDLINE(R) ALL <1946 to October 18, 2021>**

| **#** | **Searches** | **Results** |
| --- | --- | --- |
| 1 | cannabis/ | 10701 |
| 2 | exp “Marijuana Use”/ | 6535 |
| 3 | Hempa/ | 102 |
| 4 | (Cannabis or Marihuana* or Marijuana* or Hemp or Hemps or Ganja or Ganjas or Hashish or Hashishs or Bhang or Bhangs or Smoking Blunt*). ti. | 17748 |
| 5 | (Cannabis or Marihuana* or Marijuana* or Hemp or Hemps or Ganja or Ganjas or Hashish or Hashishs or Bhang or Bhangs or Smoking Blunt*). ab. /freq=3 | 13192 |
| 6 | (weed* adj3 (smok* or use* or using or abus* or dependen* or disorder*)). tw. | 629 |
| 7 | (hempa or hmpa or Hexametapol or Hexamethylphosphor*). tw. | 500 |
| 8 | 4 or 5 or 6 or 7 | 21684 |
| 9 | limit 8 to (“in data review” or in process or publisher or “pubmed not medline”) | 3794 |
| 10 | 1 or 2 or 3 or 9 | 19770 |
| 11 | adolescent/ | 2129365 |
| 12 | (adolesc* or juvenile* or teen* or youth* or pubescen* or preadolesc* or prepubesc* or preteen* or tween* or (young* adj3 (adult* or male? or female? or wom? n or lady or ladies or man or men or person? or people)) or ((emerging or early) adj1 (adult* or male? or female? or wom? n or lady or ladies or man or men or person? or people)) or (work* adj2 age?)). tw. | 713686 |
| 13 | 11 or 12 | 2490810 |
| 14 | adult/ or young adult/ | 5379127 |
| 15 | adult*. tw. | 1371813 |
| 16 | 14 or 15 | 6181493 |
| 17 | 13 and 16 | 1703663 |
| 18 | age of onset/ | 39615 |
| 19 | age factors/ | 466913 |
| 20 | (age adj3 (factor or initiation or onset)). tw. | 53153 |
| 21 | 17 or 18 or 19 or 20 | 2099999 |
| 22 | 10 and 21 | 4368 |
| 23 | ((adverse or deleter* or negative or harmful or acute) adj5 (effect* or outcome* or result* or impact* or symptom*)). tw. | 719458 |
| 24 | Depressive Disorder/ | 74254 |
| 25 | Depression/ | 133165 |
| 26 | exp Mental Disorders/ | 1323121 |
| 27 | Anxiety/ | 91723 |
| 28 | exp Psychotic Disorders/ | 54757 |
| 29 | substance-related disorders/ or psychoses, substance-induced/ | 104229 |
| 30 | schizophrenia/ or schizophrenia, paranoid/ | 108304 |
| 31 | Marijuana Abuse/ | 6684 |
| 32 | or/24–31 | 1445365 |
| 33 | (ae or de or pp). fs. | 6102145 |
| 34 | 32 and 33 | 355452 |
| 35 | 23 or 34 | 1048596 |
| 36 | 22 and 35 | 1113 |
| 37 | limit 36 to (english or french) | 1061 |
| 38 | (Animals/ or Models, animal/ or Disease models, animal/) not Humans/ | 4866238 |
| 39 | ((animal or animals or canine* or cat or cats or dog or dogs or feline or hamster* or lamb or lambs or mice or monkey or monkeys or mouse or murine or pig or pigs or piglet* or porcine or primate* or rabbit* or rats or rat or rodent* or sheep* or veterinar*) not (human* or patient* or women or men)). ti, kf, jw. | 2468832 |
| 40 | 38 or 39 | 5304319 |
| 41 | 37 not 40 | 1052 |
| 42 | limit 41 to (case reports or comment or congress or editorial or interview or introductory journal article or lecture or letter or news or newspaper article or overall or patient education handout) | 52 |
| 43 | 41 not 42 | 1000 |
| 44 | limit 43 to abstracts | 910 |
| 45 | limit 44 to dt=20201027-20211020 | 66 |
